# MATISSE: a method for improved single cell segmentation in imaging mass cytometry

**DOI:** 10.1186/s12915-021-01043-y

**Published:** 2021-05-11

**Authors:** Matthijs J. D. Baars, Neeraj Sinha, Mojtaba Amini, Annelies Pieterman-Bos, Stephanie van Dam, Maroussia M. P. Ganpat, Miangela M. Laclé, Bas Oldenburg, Yvonne Vercoulen

**Affiliations:** 1 Molecular Cancer Research, Center for Molecular Medicine, University Medical Center Utrecht, Utrecht University, 3584 CX Utrecht, The Netherlands; 2 Oncode Institute, Utrecht, The Netherlands; 3 Department of Pathology, University Medical Center Utrecht, Utrecht University, 3584 CX Utrecht, The Netherlands; 4 Department of Gastroenterology and Hepatology, University Medical Center Utrecht, Utrecht University, 3584 CX Utrecht, The Netherlands

**Keywords:** Imaging mass cytometry, Microscopy, Single cell segmentation, Immune histochemistry, Colorectal tissue

## Abstract

**Background:**

Visualizing and quantifying cellular heterogeneity is of central importance to study tissue complexity, development, and physiology and has a vital role in understanding pathologies. Mass spectrometry-based methods including imaging mass cytometry (IMC) have in recent years emerged as powerful approaches for assessing cellular heterogeneity in tissues. IMC is an innovative multiplex imaging method that combines imaging using up to 40 metal conjugated antibodies and provides distributions of protein markers in tissues with a resolution of 1 μm^2^ area. However, resolving the output signals of individual cells within the tissue sample, i.e., single cell segmentation, remains challenging. To address this problem, we developed MATISSE (iMaging mAss cyTometry mIcroscopy Single cell SegmEntation), a method that combines high-resolution fluorescence microscopy with the multiplex capability of IMC into a single workflow to achieve improved segmentation over the current state-of-the-art.

**Results:**

MATISSE results in improved quality and quantity of segmented cells when compared to IMC-only segmentation in sections of heterogeneous tissues. Additionally, MATISSE enables more complete and accurate identification of epithelial cells, fibroblasts, and infiltrating immune cells in densely packed cellular areas in tissue sections. MATISSE has been designed based on commonly used open-access tools and regular fluorescence microscopy, allowing easy implementation by labs using multiplex IMC into their analysis methods.

**Conclusion:**

MATISSE allows segmentation of densely packed cellular areas and provides a qualitative and quantitative improvement when compared to IMC-based segmentation. We expect that implementing MATISSE into tissue section analysis pipelines will yield improved cell segmentation and enable more accurate analysis of the tissue microenvironment in epithelial tissue pathologies, such as autoimmunity and cancer.

**Supplementary Information:**

The online version contains supplementary material available at 10.1186/s12915-021-01043-y.

## Background

Multiplex imaging technologies have revolutionized our ability to study cellular heterogeneity in tissues. These methods allow visualization of spatial organization of the tissue and quantification of different cell types. Moreover, these methods can yield precise views of cell-to-cell differences, quantify differences in signaling status, map signaling network topologies, and lead to important mechanistic insights. Visualizing and quantifying cellular heterogeneity in tissue samples is of increasing importance in many areas of biology, most notably in cancer research and oncology, where we now appreciate that the heterogeneous tumor microenvironment has profound implications for study, diagnosis, prognosis, and treatment of cancer [1].

The most commonly used multiplex imaging technologies fall into two main categories: microscopy based multiplex immune-histochemistry (IHC) methods [2, 3] and mass spectrometry-based methods [4], including imaging mass cytometry (IMC) [5]. IMC uses metal conjugated antibodies to label specific protein markers in a given tissue section followed by laser ablation, which allows for analysis of 1 μm^2^ tissue area at a time. Cytometry time-of-flight (CyTOF) mass spectrometry is then used to analyze metal isotope distribution as a readout for protein markers. Unlike fluorescence-based microscopy imaging methods where only 4 or 5 markers can be labeled and visualized simultaneously, mass spectrometry-based imaging methods can provide simultaneous labeling and readout of approximately 40 markers. However, one of the main challenges for mass spectrometry-based imaging methods, such as IMC, is the difficulty of single cell segmentation, i.e., distinguishing signals coming from individual cells. Currently available pipelines, such as an ilastik [6] and CellProfiler [7] extension that allows segmentation of IMC data [8], use membrane, cytosolic, and nuclear markers for single cell segmentation. These strategies are limited by the intrinsic IMC 1 μM pixel size resolution, making it difficult to discriminate cells in densely packed areas, such as epithelial layers, or immune cell infiltrates. This can often result in erroneous interpretation of nuclear and membrane signals by either merging of multiple cells into one event, or fragmenting single cells into multiple events, causing inaccuracies in these analyses.

**Fig. 1:**
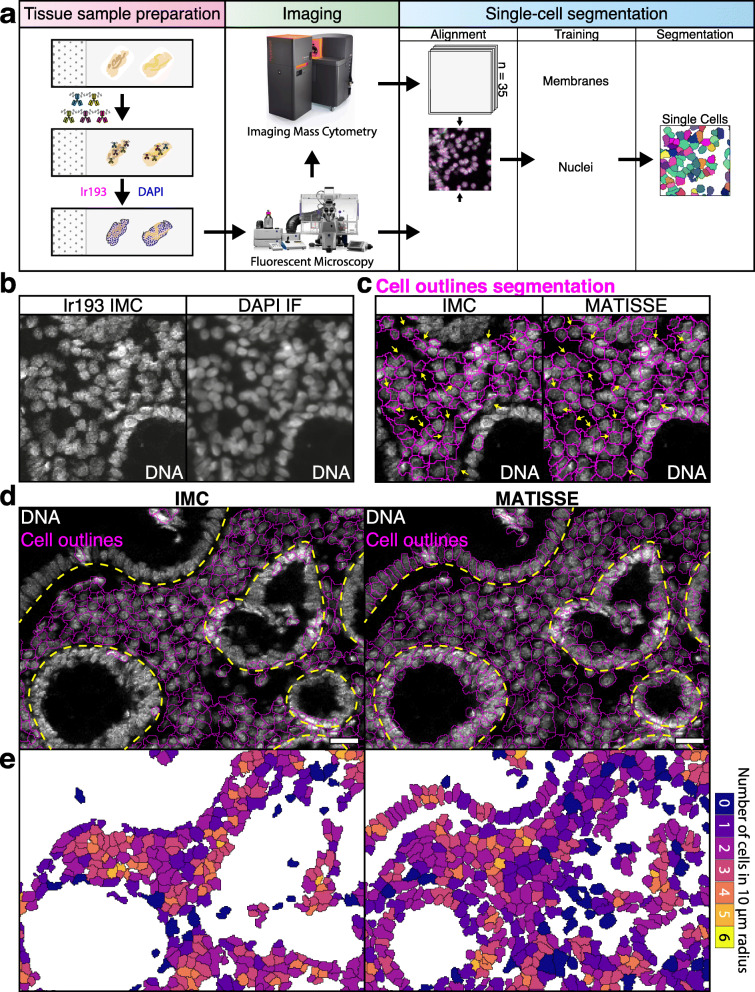
Combining fluorescence microscopy with multiplex IMC data of colorectal tissue advances quality of single cell segmentation. **a** Cartoon describing MATISSE, a novel pipeline adding microscopic imaging to multiplex IMC analysis and downstream segmentation. In short: tissue sections on slides were stained using isotope-conjugated primary antibodies, DNA intercalator, and DAPI. The tissue was first scanned using a fluorescent microscope and then processed with IMC. Data produced by both techniques is aligned using the nuclear staining. Nuclear and membranous pixel probability maps are produced based on the fluorescent images and IMC data respectively. These probability maps are used to generate a segmentation map, where all detected cells are included. **b** Representative images of DNA intercalator on a colorectal tissue section analyzed by Ir193 labeling and IMC (left) or DAPI labeling and fluorescent microscopy (IF, right). **c** IMC-only (IMC) and MATISSE cell segmentation (MATISSE) were performed, and shown are the different predicted outlines on a representative image of Ir193 labeling. Arrows indicate areas with cell fragmentation. **d** Display of a large region of interest (ROI) showing an overlay of the predicted cell outlines (pink) upon IMC or MATISSE segmentation on a representative IMC image of DNA-Ir193 labeling of colorectal tissue. Highlighted in yellow is the approximate position of the basement membrane surrounding the epithelial monolayer. Scale bar 25 μm. **e** Cell density was calculated as the number of cells within a radius of 10 μM from the center of each single cell [9, 10]. This number is displayed with a color code for each cell in the representative image

While IMC resolution is limited by a fixed 1 μM pixel size, fluorescent microscopy allows acquisition at variable resolutions well below 1 μM pixel size. Therefore, to provide a solution for the single cell segmentation problem in IMC, we have developed MATISSE (iMaging mAss cyTometry mIcroscopy Single cell SegmEntation, Fig. 1a), a method that combines the use of IMC and fluorescent microscopy imaging into a single workflow. More specifically, we designed MATISSE to use a multiplex IMC antibody panel containing membrane, cytoplasm, and nuclear markers, as well as fluorescent nuclear DAPI and DNA intercalator labeling of the same tissue region for improved segmentation. The MATISSE tissue analysis pipeline begins with staining tissue sections with metal isotope conjugated antibodies, DNA intercalator, and DAPI, followed by fluorescence microscopy and IMC. The data obtained from two imaging techniques is then aligned using nuclear staining, and pixel probability maps for membranes and nuclei are calculated based on IMC and DAPI data, respectively. These probabilities are combined into a single segmentation map that is representative of the cells in the tissue section. We have developed MATISSE based on existing technologies and used open-access tools to create scripts for automated alignment of the IMC and IF datasets, as our goal was to produce a method that can be readily implemented by other research laboratories (see Additional file 1: MATISSE_MANUAL).

## Results

To test and validate the performance of the MATISSE pipeline, we benchmarked it against the current standard in the field, the IMC-only segmentation pipeline (IMC) [8]. We used colorectal biopsy sections from patients with different stages of inflammatory bowel disease (IBD) as our tissue of choice, given the established heterogeneity of IBD clinical phenotypes and challenges this poses when diagnosing and treating patients [11]. Recent studies have revealed a rewiring of both intracellular signaling and cellular interactions between intestinal epithelium, stroma, and immune cells in IBD patients [12], and significant colonic epithelial cell diversity [13], further highlighting the need for more accurate strategies for quantitative analysis at a single cell level, which would be ultimately applicable to multiple different types of tissues.

**Fig. 2:**
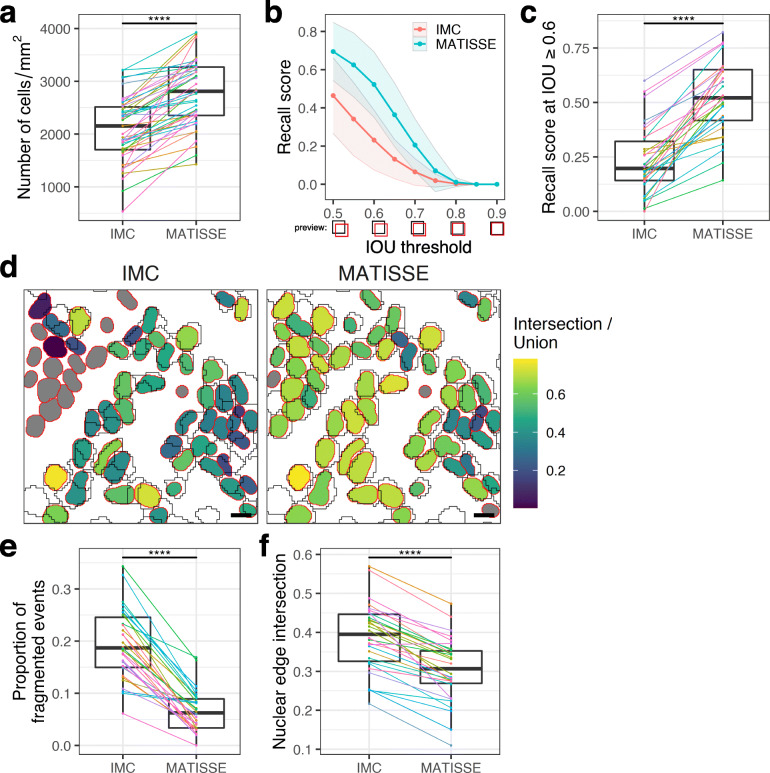
MATISSE segmentation promotes both cell identification quantity and quality. **a** Numbers of cells were quantified using IMC and MATISSE segmentation methods for all analyzed regions of interest (ROIs). Lines link the datapoints per ROI. Paired *t* test was performed to test for significance. ****p < 0.0001. *N* = 45 images. **b**, **c** Overlap between manual annotations and predictions was quantified by recall score and **b** compared for MATISSE and IMC at varying intersection-over-union (IOU) thresholds, **c** displayed per ROI at IOU 0.6 and higher, lines link datapoints per ROI. Paired *t* test was performed to test for significance. ****p < 0.0001. *N* = 30 images. **d** Representative image of IOU values indicated by a color-scale labeling of the annotated events (red lining) that overlap with predictions by IMC or MATISSE. Black lines indicate outlines of the predictions. Scale bar 25 μm. **e** Fraction of split annotated events were quantified using IMC and MATISSE segmentation methods for all ROIs, lines link the datapoints per ROI. Paired *t* test was performed to test for significance. ****p < 0.0001. *N* = 30 images. **f** Edge intersection score per ROI was determined by quantifying intersection of predicted cell outlines by both methods with manually annotated nuclei, where a lower score corresponds to less overlap. Lines link the datapoints per ROI. Paired t-test was performed to test for significance. ****p < 0.0001. *N* = 30 images

Here, we prepared the tissue sections according to the procedure described in the “Methods” section (see also Additional file 2: Table 1, Additional file 3: Fig. S1A), and we performed pixel classification using the machine learning tool Ilastik [6]. Experienced users were tasked to generate training data for nuclear and membrane markers. Specifically for nuclear fluorescent DAPI signal Fiji [14] was used to generate annotations for training. Primary (nuclei) and secondary (cells) objects were identified with CellProfiler [7] based on the probability maps generated by Ilastik (for a detailed overview of the full procedure, see the “Methods” section, and additional file MATISSE_MANUAL). We observed that incorporating fluorescent microscopy images based on DAPI nuclear staining into MATISSE workflow resulted in superior visual and signal intensity-based separation of nuclei in dense areas (Fig. 1b, Additional file 3: Fig S1B). Next, we assessed segmentation maps generated by both segmentation methods. The predicted cell outlines differed between IMC and MATISSE methods in colon (Fig. 1c, d), small intestine, and more different tissues such as skin, liver, and non-small cell lung cancer (Additional file 3: Fig. S1C). Quantitative analysis emphasized improved cell segmentation by MATISSE, which identified significantly higher numbers of cells in all regions of interest (ROIs) (Fig. 2a, IMC mean 2086 ± 611 S.D., MATISSE mean 2783 ± 622 S.D.), and an improved recall score at different rates of overlap between predicted outlines and annotated ground (intersection over union) (Fig. 2b–d, Additional file 3: Fig. S2B) [15]. Of note, DNA IMC signal intensity in single cells between images was similar (Additional file 3: Fig. S2C). Moreover, MATISSE displayed less cell fragmentation, as shown by a comparative analysis of cell density (Fig. 1e), decreased fraction of fragmentation events (Fig. 2e, Additional file 3: Fig. S2A), and improved edge intersection score (Fig. 2f), indicating that the increased cell number was not caused by erroneous fragmentation of cells. Together, this comparative analysis showed that MATISSE resulted in both a superior quality of segmentation, and identification of a larger number of cells in the tissue.

**Fig. 3:**
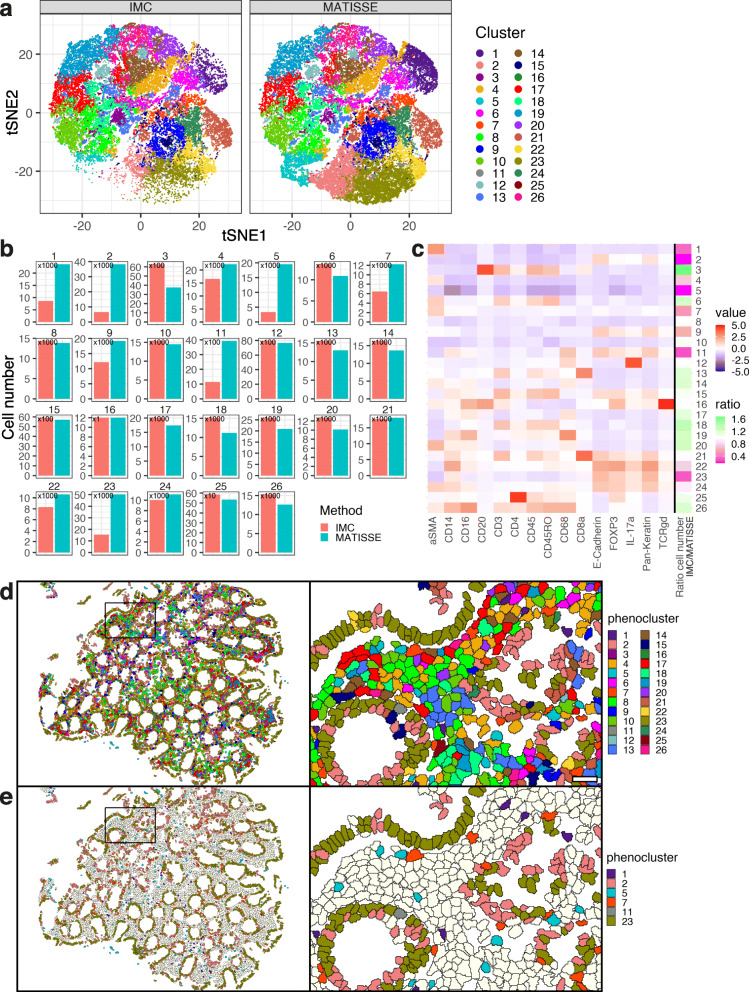
MATISSE improves identification of specific cell subsets in colorectal tissue. **a** Forty-five regions of interest (ROIs) in 10 different tissue sections were imaged and segmented using IMC or MATISSE pipelines, and 10% of all identified single cells were included in a t-SNE. Twenty-six phenoclusters [16] were identified and displayed with a color-code. *N* = 29242 cells for IMC, 38430 cells for MATISSE. **b** Numbers of cells contained per cluster were calculated and displayed for both IMC and MATISSE methods. *N* = 45 images. *N* = 291919 cells for IMC, 384804 cells for MATISSE. **c** Heatmap display of the mean signal intensity per cell in each cluster. The ratio of number of cells identified by both segmentation methods (IMC / MATISSE) per cluster is displayed on the right. *N* = 45 images. **d**, **e** Spatial location of single cells in the tissue was visualized and color-coded by phenocluster. Displayed are overview images of an entire ROI (left) and a zoom of a specific region (right), shown are all phenoclusters (**d**), top 6 differential phenoclusters with higher cell numbers in MATISSE (**e**). Scale bar 25 μm

Given the differences in numbers and segmentation quality of identified cells, we next set out to examine which cell types or tissue regions were differently segmented and thus most impacted by an improved segmentation pipeline. Clustering analysis was performed on all single cell events of all included ROIs combined to assess identified cell types, resulting in 26 clusters represented in a t-SNE plot (Fig. 3a, see Additional file 4: Table 2). Comparison of the number of cells identified in each cluster showed that specific clusters were affected by the method of segmentation in multiple ROIs (Fig. 3b, Additional File 3: Fig S3A), confirming that improved segmentation leads to differences in quality and quantity for downstream analysis of the data. Multiple clusters displayed differences in cell numbers, including clusters with low membrane signal in IMC, and clusters displaying clear positive signal in multiple channels, indicating that signal intensity of a specific population did not bias the observed differences in segmentation (Fig. 3c). The 6 clusters with largest increase of cell numbers in MATISSE versus IMC included fibroblasts (clusters 1 and 7), epithelial cells (clusters 2, 11, 23), myeloid cells and intra-epithelial lymphocytes (clusters 11 and 23), and negative cells expressing no significant levels of stained markers (cluster 5). Next, we visualized all single cells at their spatial location in the tissue, color-coded by cluster number (Fig. 3d, Additional file 3: Fig S3B). Focusing on the 6 clusters displaying the largest increase in cell numbers using MATISSE showed localization throughout the tissue, as expected, with clusters 2 and 23 locating in the epithelial layer, clusters 1, 7, and 11 in the basal membrane just below the epithelium, and clusters 1, 5, and 11 in the lamina propria (Fig. 3e, Additional file 3: Fig S3C). The 6 clusters that showed lower cell numbers in MATISSE versus IMC, and 7 clusters with equal numbers of cells in both segmentation methods, were analyzed in a similar fashion (Additional file 3: Fig S3C). This highlighted that cells identified with both methods can occur at similar spatial locations but appear more often fragmented (Additional file 3: Fig. S3D) or differently clustered in several examples using IMC-based segmentation compared to MATISSE.

## Discussion

We show that the segmentation maps between IMC-based and MATISSE methods displayed major differences. The differences were most pronounced for specific cell populations, such as epithelial cells, fibroblasts, and specific immune cells where using higher resolution data in MATISSE facilitated improved annotation of separate nuclei and, consequently, superior training and segmentation. Rendering improved segmentation maps with MATISSE led to clear quantitative and qualitative changes in downstream analysis. Moreover, MATISSE demonstrated improved segmentation in the small intestine, which is similar to colon, but also in tissues such as skin and liver and in samples from non-small cell lung cancer. Of note, the panel and training in this study were designed for analysis of colon tissue and still showed reasonable performance. Future studies examining whether targeted training would even further improve performance in a range of tissues are warranted. For optimal accessibility, MATISSE has been developed based on existing technologies and open-access tools and can therefore be readily applied to different tissues and IMC antibody panels.

## Conclusions

Taken together, MATISSE allowed segmentation of cellular areas such as the colonic mucosa, and keratinocyte layer of the skin, and showed a qualitative and quantitative difference in the outcome of analysis compared to IMC-based segmentation. Going forward, we expect that implementing MATISSE into tissue section analysis pipelines of colorectal samples, and beyond, will yield improved cell segmentation and enable more accurate analysis of the tissue microenvironment in tissue pathologies, such as autoimmunity and cancer.

## Methods

### Patients

Historical formalin-fixed paraffin embedded (FFPE) tissue blocks of colonic biopsies from patients with inflammatory bowel disease (IBD) were collected and included. Informed consent was obtained from all patients. Ten tissue sections were included from 3 patients with IBD, of 3 separate timepoints of biopsy per patient. The study was approved by the medical ethical board of the UMC Utrecht (METC protocol #11-050/E, and biobank protocols #18-676). Furthermore, TMA sections of rest-material, including intestine, liver, and non-small cell lung cancer, were included according to the no-objection-agreement, approved by the UMC Utrecht biobank committee (protocol #18-222).

### Antibodies and reagents

For a comprehensive list of antibodies, compounds, and kits, see Supplementary Table 1.

### Sample preparation

Tissue sections of 4 μM thickness were cut and placed on a glass slide (brand, specifics).

Slides were baked for an hour at 60 °C. Samples were deparaffinized in xylene twice for 10 minutes, followed by rehydration in a graded series of ethanol (100% 10 min, 95% 5 min, 80% 5 min, 70% 5 min), washed in Milli Q water 3 min, and finally PBST (TBS containing 0.1% Tween) for 10 min. Antigen retrieval was performed for 30 min at 96 °C in Tris-EDTA (10 mM Tris, 1 mM EDTA) pH 9, followed by a cool-down period of 10 min at room temperature. Samples were incubated in TBST for 10 min. Tissue sections were encircled using a PAP pen. Blocking was performed with TBST containing 3% BSA and FC-block 1:100 for 1 h at room temperature. Metal-conjugated antibodies were diluted according to dilutions stated in supplementary Table 1 in TBST containing 0.5% BSA. Staining was performed in a humidified chamber overnight at 4 °C. Samples were then washed in TBST twice for 5 min and TBS for 10 min, followed by incubation with 300 times diluted DNA intercalator Ir193 (Fluidigm) and 1000 times diluted DAPI in PBS for 1 h at room temperature. Then, slides were then washed twice with ddH_2_O for 5 min. Samples were mounted in 90% glycerol and covered with a coverslip for microscopy.

### Fluorescent microscopy imaging

Slides were imaged on a Zeiss CellObserver using a × 20 dry objective (0.75 NA, 420150-9900). A Colibri 7 was used as light source, in combination with a Zeiss 90 HE filter set. The system was equipped with a Hamamatsu Orca Flash4.0 V2+ camera (C11440-22CU). Images were acquired in a tiled Z-stack format with 10% overlap between tiles and 9 Z-slices using ZEN software (2.3). ZEN was again used to export imaging data to individual 16-bit tiff tiles. Z-stacks were converted to single in-focus images using the Extended Depth of Field plugin in Fiji at highest quality settings [14, 17]. Tile images were stitched using the MIST algorithm in Fiji [18].

### Mass cytometry imaging

After microscopy, samples were unmounted by dipping and washing in ddH_2_O. Samples were stained with toluidine blue for 5 min at RT, washed for 3 min with ddH_2_O, and dried. Mass cytometry imaging was performed on a Hyperion (Fluidigm) laser ablation module, coupled to a Helios (Fluidigm) mass cytometer. Tuning was performed according to manufacturer instructions. Laser ablation frequency was set to 200 Hz. Data files were converted to 32-bit tiff files using the imctools library (https://github.com/BodenmillerGroup/imctools).

Histone H3 imaging results typically show nuclear localization with a different staining pattern compared to DNA intercalator (Ir193, and DAPI), since they bind to different targets, and Histone H3 is an antibody staining. Therefore, we used both intercalators for registration (see below).

### Registration

Fluorescent microscopy images of DAPI were registered to mass cytometry images of the DNA-intercalator signal, while retaining the resolution of the fluorescent images. Registration was performed using key points generated using the MOPS algorithm [19]. Images were transformed using the landmark correspondence command in Fiji (https://imagej.net/Landmark_Correspondences). The method for transformation used was moving least squares and transformation class similarity.

### Profile plot

The plot profile function in Fiji [14] was used to determine signal intensity along lines. Images of DAPI and DNA-intercalator were co-registered, but analysis was performed at original resolution. Intensity values were normalized per line and marker.

### Probability map generation mass cytometry images

Before training on imaging mass cytometry data, signal intensity was manually scaled and converted to a 16-bit range. Annotations were generated by experienced users using Ilastik [6] (1.3.3). The following channels were selected for machine learning: Pan-Keratin, E-Cadherin, αSMA, Histon H3, 193Ir, LMNB1, Ki-67, CD3, CD4, CD8a, CD14, CD16, CD20, CD45, CD45RO, and CD68.

Training data was generated for 5 classes, namely non-epithelial cellular membranes, non-epithelial nuclei, epithelial cytoplasm/membrane, epithelial nuclei, and background. Training and probability map generation was performed in Ilastik using only IMC data as input. In Ilastik all features with sigma between 0.3 and 1.6 were selected. Probability maps were saved to individual 32-bit tiff files for each class.

### Probability map generation fluorescent microscopy images

Training data was generated on a random subset of 100 tile regions of fluorescent imaging data. Annotations were made by 4 experienced users for classes nuclei, edges of nuclei, and background, using Fiji [14] based only on DAPI signal. No contrast adjustments were made. Feature images and morphological filters of the raw DAPI signal were made using FeatureJ [20] and MorphoLibJ [21] respectively and added as channels to the imaging data before training in Ilastik. Features: Laplacian (σ 0.7, 1, 1.6, 2, 3), Hessian-smallest (σ 3, 5), Hessian-largest (σ 1, 2), Structure-largest (σ 1, 2), Gaussian (σ 0.7, 1.6, 2, 3.5). Morphology filters: Opening (σ 1, 2, 3, 5), Internal Gradient (σ 1, 3, 5), White Top Hat (σ 8, 10, 15, 20), Edges (σ 1, 2). In Ilastik, only the Gaussian smoothing feature with sigma 1.0 was selected, since all feature images are included in the input data. Probability maps were generated for stitched tile-scan images. Probability maps were saved to individual 32-bit tiff files for each class. Ilastik was used in headless mode on a high-performance computer cluster using 8 cores and 100 GB of memory.

### Single cell segmentation

Segmentation was performed using Cellprofiler [7] (v3.1.9). The pipeline can be found in the online methods. For both segmentation approaches, cells were identified firstly by identification of individual nuclei and secondly by expansion of these nuclei to the full extent of the cells. The segmentation map was stored in a 16-bit tiff format.

For IMC-based segmentation, only the probability maps generated using the IMC-data were used. For MATISSE segmentation, identification of nuclei was based on the probability maps generated using the fluorescent images of DAPI, at high resolution. The identified nuclei were next downscaled to the resolution of the IMC data and expanded to the full extent of the cells using the membrane probability generated using only the IMC data.

### Segmentation score

#### Manual annotations for ground truth

Trained experts were asked to manually annotate individual nuclei in a subset of 30 images of nuclear staining (100 × 100 μm). These images were a composite of both fluorescent and IMC data, of DAPI and DNA-intercalator respectively, after co-registration, at high resolution. In total 2642 nuclei were annotated. The annotations were converted to a binary mask and downscaled to the resolution of IMC data.

### Segmentation score calculation

For calculation of a recall score, first the intersection over union (IOU) was calculated. The IOU is determined for all manually annotated nuclei that overlap with any nuclear outline generated by either segmentation pipeline separately. IOU is calculated as the surface area of the intersection area between nuclear outlines, divided by the surface area of the union of both nuclear outlines. For each manual event only the interaction with highest IOU was taken for recall calculation, in case of multiple identified overlaps [15]. Recall is calculated as the number of true positive events, divided by the sum of number of true positive events and number of false negative events. Specifically, this recall is calculated at different required IOU thresholds, ranging from 0.5 to 1.0 with increments of 0.05.

The proportion of split events is calculated as the fraction of ground truth nuclear annotations that overlap with multiple nuclear events from either segmentation pipeline, over the total number of events that have any overlap. Overlap being defined as at least 20% of surface area of the ground truth object.

A probability score was calculated for intersection of cell boundaries, derived from segmentation maps from automated pipelines, with manually annotated nuclei [22]. This was performed for both IMC-based and MATISSE segmentation methods.

For calculation of fragmentation per phenocluster, we calculated the proportion of events generated by either segmentation pipeline that was identified as part of a fragmentation event, from the total number of events that overlap for at least 20% with any ground truth annotation. Fragmentation events being at least two events overlapping with a single ground truth annotation for at least 20% of the ground truth surface area per interaction.

### Single cell data generation

Single cell data was generated in R (v4.0) [23] by extracting pixel intensities from unscaled 32-bit images for all channels for all cells represented in the segmentation maps for both IMC-only and MATISSE methods.

### Spatial analysis

Segmentation maps were converted to polygons in R using packages sf [9] and stars [24]. Distances between neighboring cells were calculated using the RANN package [10] based on centroids of the cells determined with the sf package. A radius of 10 μm was used to count the number of direct neighbors for each cell and used as a measure for density.

### Clustering

Single cell clusters were generated with the Rphenograph package [16], based on the mean expression per cell of the markers αSMA, CD14, CD16, CD20, CD3, CD4, CD45, CD45RO, CD68, CD8a, E-Cadherin, FOXP3, IL-17α, Pan-Keratin, and TCRγδ. This clustering was performed using pooled single cell data generated by both segmentation methods. The number of nearest neighbors was kept at the default value of 30 for clustering. tSNE was performed using the Rtsne package [25–27], again using pooled data, with settings initial dimensions 50, perplexity 30, and theta 0.5. Single cell data was log1p transformed for clustering and tSNE.

### Phenocluster spatial representation

Polygons representing cell outlines were plotted and given a random color fill corresponding to their assigned phenocluster number. Clusters with a large difference in number of cells across segmentation methods were selected by taking the top 6 with the highest or lowest ratio. Clusters assumed to be equally represented in both segmentation methods were selected based on a ratio between 0.9 and 1.1. Random regions were generated by selecting a region of 100 by 100 μm from 4 randomly selected ROIs.

## Supplementary Information

**Additional file 1.** MATISSE_MANUAL: an interactive PDF outlining all steps of computational methods, to allow smooth implementation of MATISSE methods. All code required is publicly accessible in Github: https://github.com/VercoulenLab/MATISSE-Pipeline.

**Additional file 2: Supplementary Table 1.** Antibody panel resources

**Additional file 3: Figure S1.** a) Representative examples of IMC images, b) nuclear staining profiles DAPI versus Ir193, and c) predicted cell outlines of different tissues. **Figure S2.** a) Representative example of overlap between manual annotations and predictions, b) Recall scores calculated for different tissues, and c) Ir193 signal intensity across all analyzed images. **Figure S3.** Comparison of IMC and MATISSE performance per phenocluster. a) Cell numbers identified per phenocluster across all analyzed images. b) representative examples of cell outlines, density and phenoclusters, c) representative examples of cells colored by specific phenoclusters, d) fragmentation events per phenocluster.

**Additional file 4: Supplementary Table 2.** Phenocluster names.

## Data Availability

All data generated or analyzed during this study are included in this published article and its supplementary information files and publicly available repositories: Datasets: Image and other processed data are publicly available on Zenodo, doi: 10.5281/zenodo.4727873 (https://zenodo.org/record/4727873). Scripts: https://github.com/VercoulenLab/MATISSE-Pipeline)
