# Publisher Correction to: MATISSE: a method for improved single cell segmentation in imaging mass cytometry

**DOI:** 10.1186/s12915-021-01065-6

**Published:** 2021-06-18

**Authors:** Matthijs J. D. Baars, Neeraj Sinha, Mojtaba Amini, Annelies Pieterman-Bos, Stephanie van Dam, Maroussia M. P. Ganpat, Miangela M. Laclé, Bas Oldenburg, Yvonne Vercoulen

**Affiliations:** 1 Molecular Cancer Research, Center for Molecular Medicine, University Medical Center Utrecht, Utrecht University, 3584, CX Utrecht, The Netherlands; 2 Oncode Institute, Utrecht, The Netherlands; 3 Department of Pathology, University Medical Center Utrecht, Utrecht University, 3584, CX Utrecht, The Netherlands; 4 Department of Gastroenterology and Hepatology, University Medical Center Utrecht, Utrecht University, 3584, CX Utrecht, The Netherlands

**Publisher Correction to: BMC Biol 19, 99 (2021)**

**https://doi.org/10.1186/s12915-021-01043-y**

Following publication of the original article [1], it was noted that due to a figure processing error during typesetting part of the image in Fig. 1a was omitted. The correct Fig. 1 has been included in this Publisher Correction, and the original article has been corrected.

**Fig. 1:**
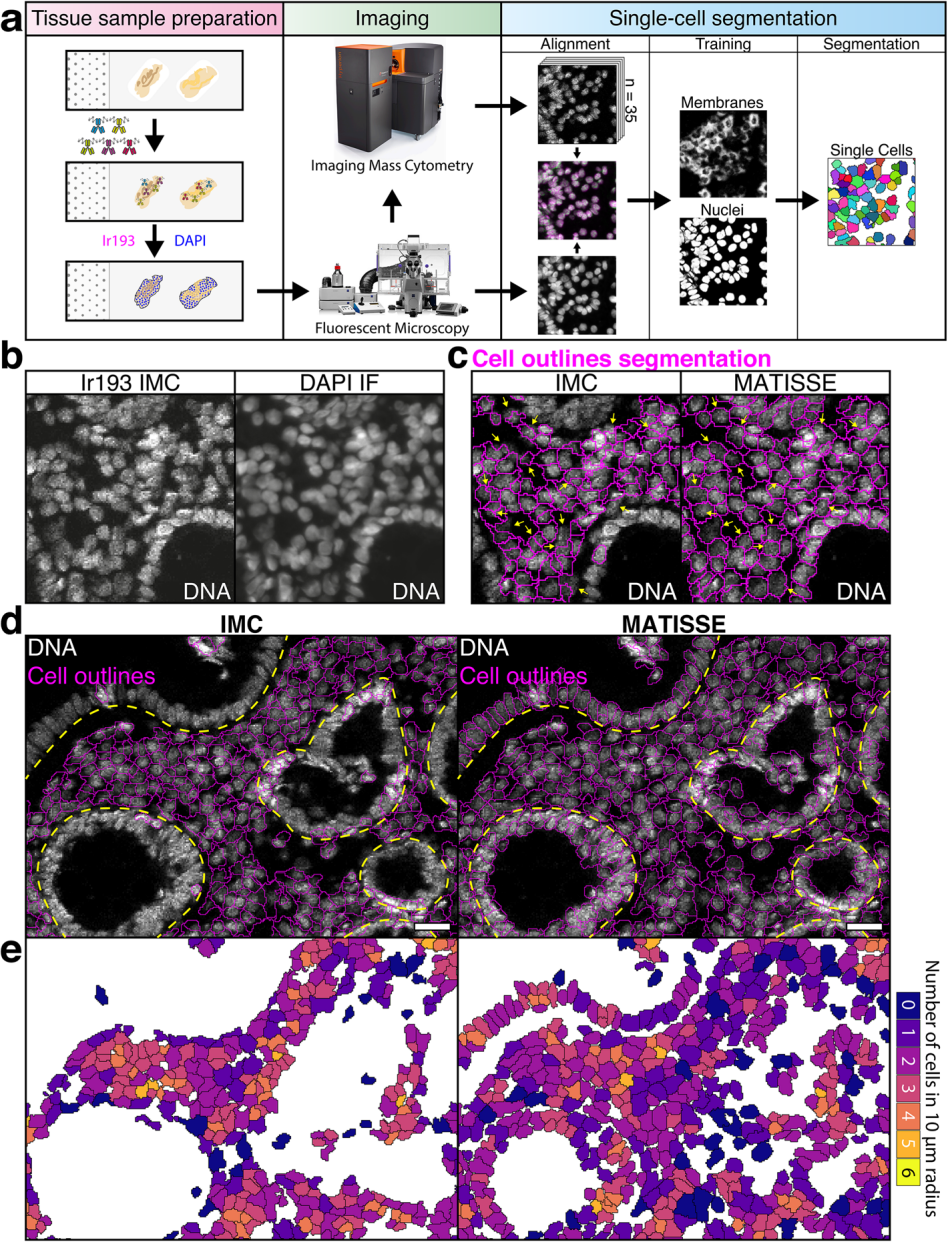
Combining fluorescence microscopy with multiplex IMC data of colorectal tissue advances quality of single cell segmentation. **a** Cartoon describing MATISSE, a novel pipeline adding microscopic imaging to multiplex IMC analysis and downstream segmentation. In short: tissue sections on slides were stained using isotope-conjugated primary antibodies, DNA intercalator, and DAPI. The tissue was first scanned using a fluorescent microscope and then processed with IMC. Data produced by both techniques is aligned using the nuclear staining. Nuclear and membranous pixel probability maps are produced based on the fluorescent images and IMC data respectively. These probability maps are used to generate a segmentation map, where all detected cells are included. **b** Representative images of DNA intercalator on a colorectal tissue section analyzed by Ir193 labeling and IMC (left) or DAPI labeling and fluorescent microscopy (IF, right). **c** IMC-only (IMC) and MATISSE cell segmentation (MATISSE) were performed, and shown are the different predicted outlines on a representative image of Ir193 labeling. Arrows indicate areas with cell fragmentation. **d** Display of a large region of interest (ROI) showing an overlay of the predicted cell outlines (pink) upon IMC or MATISSE segmentation on a representative IMC image of DNA-Ir193 labeling of colorectal tissue. Highlighted in yellow is the approximate position of the basement membrane surrounding the epithelial monolayer. Scale bar 25 μm. **e** Cell density was calculated as the number of cells within a radius of 10 μM from the center of each single cell [9, 10]. This number is displayed with a color code for each cell in the representative image

The publisher apologises to the authors and readers for the inconvenience caused by the error.
