# Supplementary material for: MATISSE: a method for improved single cell segmentation in imaging mass cytometry
Source: BMC Biol. 2021 May 11;19:99. doi: 10.1186/s12915-021-01043-y (PMC8114487; doi:10.1186/s12915-021-01043-y)
Supplement: Supplementary file 1 — Additional file 1. MATISSE_MANUAL: an interactive PDF outlining all steps of computational methods, to allow smooth implementation of MATISSE methods. All code required is publicly accessible in Github: https://github.com/VercoulenLab/MATISSE-Pipeline. [file 12915_2021_1043_MOESM1_ESM.pdf]

MATISSE Workflow Manual

MATISSE pipeline contains a selection of ImageJ, Cellprofiler and Ilastik modules that facilitate handling and processing of imaging data. In addition to using open access ImageJ, cell profiler & Ilastik for analysis, we have generated in house R script for data handling and visualization which are accessible online in MATISSE GitHub repository. Below are the outline of the workflow and detailed descriptions for the script/tools that were used for analysis. **Click on desired box (resembling buttons) for manual for each step.**

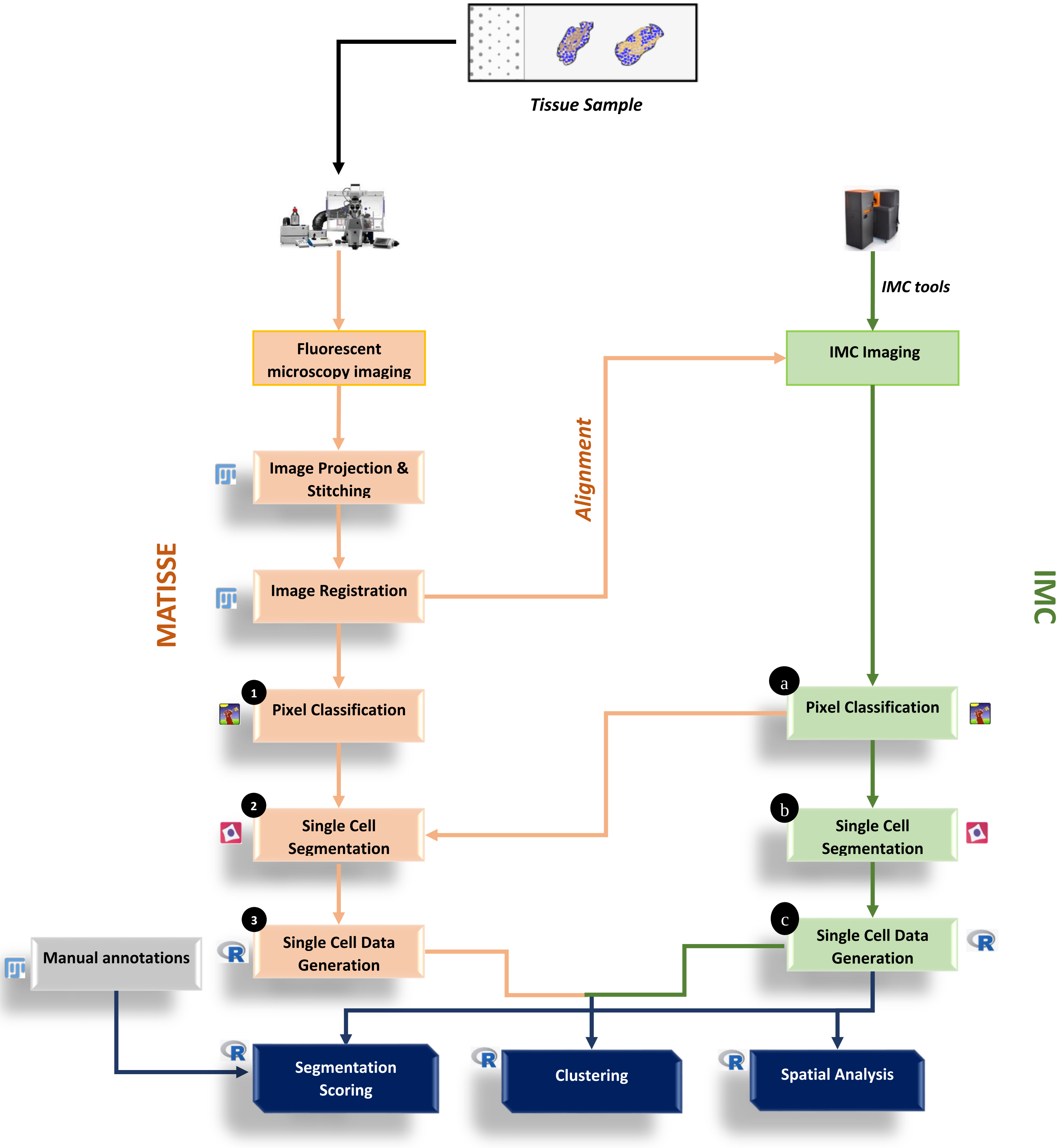

Image Projection & Stitching:

MIST plugin in Fiji was used to stitch the image. A detailed description of how to use this plugin in Fiji is provided in **MIST GitHub repository**.

Image registration:

Image registration was performed using the Landmark correspondence command in Fiji. We used plugin "Extract SIFT correspondences" (under "plugins>Feature extraction") which calculates the landmarks ("Plugins>Transform>Landmark Correspondences").

Pixel Classification:

1. MATISSE:

FeatureJ and MorphoLibJ plugins in Fiji were used for the extraction of image features and 2D segmentation of grayscale images, based on morphological operations and watershed transform respectively. We have provided script (***FilterAdditionStack.ijm***) in the MATISSE GitHub repository to use this plugin. We also have provided script (***ManualAnnotation_Training.ijm***) in the same repository to generate training data.

Ilastik was used in headless mode to generate probability maps for Fluorescent Microscopy Images.

a. IMC:

Ilastik was used in headless mode to generate probability maps for IMC.

Single cell Segmentation

2. MATISSE:

Cellprofiler (v3.1.9) was used for segmentation, and the detailed tutorial can be found in the Cellprofiler GitHub repository.

b. IMC:

Same pipeline as above of Cellprofiler (v3.1.9) was used for single cell Segmentation of IMC data.

Single cell data generation:

3. MATISSE:

We have use custom R script (***ExtractSingleCellDataParallel.R***) to generate single cell data.

c. IMC:

Same script as above (***ExtractSingleCellDataParallel.R***) was used to generate single cell data.

Manual Annotation:

ManualAnnotation_SegmentationScore.ijm script in ImageJ was used for manual annotation.

Segmentation Scoring:

SegmentationScoreNucleus.R script in R was used to generate the segmentation score.

Clustering:

PhenoGraph version_0.99.1 was used for clustering, and the detailed tutorial can be found in the

Rphenograph GitHub repository.

Spatial Analysis:

SpatialAnalysis.R script in R was used for Spatial Analysis.

Evaluation Metrics for Segmentation

Recall

Recall is the ratio of correctly predicted annotations to the total number of ground truth annotations. It effectively accounts for all of the objects annotated in ground truth, and shows how many were captured as positive predictions (TP)[1].

$$Recall = True\ positive / True\ Positive + False\ negative$$

To constitute positive detections we have calculated Intersection over Union (IoU) score between ground truth and predictions.

Intersection over Union (IoU)

The Intersection over Union (IoU) metric, also referred as Jaccard index is an evaluation metric used to quantify the accuracy of a segmentation of an image. It also allows us to evaluate how similar a prediction is to the ground truth box[2, 3].

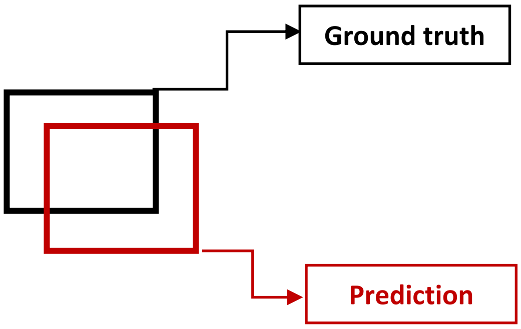

Intersection over union (IoU) defined as:

$$\boldsymbol{IoU = \frac{Ground\ truth \cap Prediction}{Ground\ truth \cup Prediction}}$$

It is very unlikely that the (x, y)-coordinates of the predicted box are going to exactly match the (x, y)-coordinates of the ground truth box, thus a complete overlap between predicted and ground truth boxes is unrealistic. For this reason, we need to define an evaluation metric that rewards predicted boxes for a high degree of overlap with the ground truth.

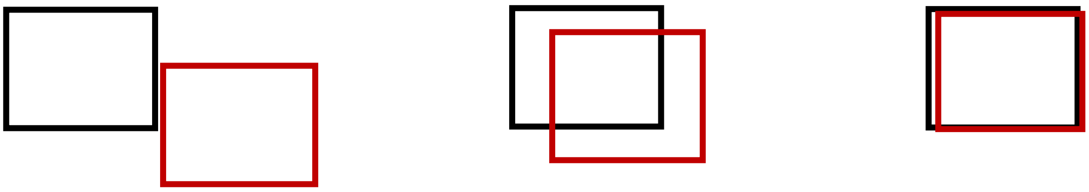

$\boldsymbol{IoU = 0}$ $\boldsymbol{IoU = 0.5}$ $\boldsymbol{IoU = 1}$

If the prediction is completely overlapping $IoU = 1$, $IoU = 0.5$ considered as good prediction and $IoU = 0$ considered as no match. In general, if an IOU score is >0.5[3], it is considered as a good threshold, however, this can vary between different datasets.

Reference

1. Intawong K, Scuturici M, Miguet S: **A New Pixel-Based Quality Measure for Segmentation Algorithms Integrating Precision, Recall and Specificity**. In*: 2013; Berlin, Heidelberg*. Springer Berlin Heidelberg: 188-195.
2. Van Valen DA, Kudo T, Lane KM, Macklin DN, Quach NT, DeFelice MM, Maayan I, Tanouchi Y, Ashley EA, Covert MW: **Deep Learning Automates the Quantitative Analysis of Individual Cells in Live-Cell Imaging Experiments**. *PLoS Comput Biol* 2016, **12**(11):e1005177.
3. Caicedo JC, Roth J, Goodman A, Becker T, Karhohs KW, Broisin M, Molnar C, McQuin C, Singh S, Theis FJ *et al*: **Evaluation of Deep Learning Strategies for Nucleus Segmentation in Fluorescence Images**. *Cytometry A* 2019, **95**(9):952-965.
