# Supplementary material for: MATISSE: a method for improved single cell segmentation in imaging mass cytometry
Source: BMC Biol. 2021 May 11;19:99. doi: 10.1186/s12915-021-01043-y (PMC8114487; doi:10.1186/s12915-021-01043-y)
Supplement: Supplementary file 2 — Additional file 2: Supplementary Table 1. Antibody panel resources [file 12915_2021_1043_MOESM2_ESM.pdf]

Supplementary Table 1

Antibody	Isotope	Clone	Supplier	Cat #	RRID	Dilution
Alpha-SMA	141Pr	1A4	Thermo Fisher Scientific	14-9760-82	AB_2572996	1:300
CD14	144Nd	EPR3653	Abcam	ab214438	N/A	1:200
CD16	146Nd	EPR16784	Abcam	ab215977	N/A	1:200
CD20	161Dy	H1	Fluidigm	3161029D	AB_2811016	1:300
CD3	170Er	polyclonal	DAKO	A045229-2	N/A	1:100
CD4	156Gd	EPR6855	Abcam	ab181724	N/A	1:100
CD45	143Nd	D9M8I	Cell Signaling Technology	13917S*	AB_2750898	1:100
CD45RO	152Sm	UCHL1	Cell Signaling Technology	55618S*	AB_2799491	1:100
CD68	159Tb	KP1	Fluidigm	3159035D	AB_2810859	1:600
CD8a	162Dy	C8/144B	Fluidigm	3162034D	AB_2811053	1:200
E-Cadherin	142Nd	24E10	Cell Signaling Technology	3195S*	AB_2291471	1:150
FOXP3	155Gd	236A/E7	Abcam	ab96048	AB_445284	1:50
Histon H3	176Yb	D1H2	Cell Signaling Technology	4499S*	AB_10544537	1:600
IL-17	167Er	polyclonal	Bio-Techne/ R&D Systems	AF-317-NA	AB_354463	1:100
Ki-67	168Er	B56	BD Biosciences	556003	AB_396287	1:200
Lamin B1	113In;115In	EPR8985(B)	Abcam	ab220797	N/A	1:100
PanKeratin	148Nd	C11	Cell Signaling Technology	4545S*	AB_490860	1:200
TCR$\gamma\delta$	174Yb	H-41	Santa Cruz	sc-100289	AB_1130061	1:50
**Reagent**	**Isotope**		**Supplier**	**Cat #**		**Dilution**
Intercalator	191/193 Ir		Fluidigm	201192B		1:300
DAPI	-		Sigma	D9542		1:1000
Xylene	-		Klinipath	4055-9005		N/A
Ethanol	-		Klinipath	4099.9005		N/A
FC-block	-		Biolegend	422302		1:100
**Antibody Labelling kit**			**Supplier**	**Cat #**		
Maxpar X8 antibody labelling kit, 141Pr			Fluidigm	**201141A**		
Maxpar X8 antibody labelling kit, 142Nd			Fluidigm	**201142A**		
Maxpar X8 antibody labelling kit, 143Nd			Fluidigm	**201143A**		
Maxpar X8 antibody labelling kit, 144Nd			Fluidigm	**201144A**		
Maxpar X8 antibody labelling kit, 146Nd			Fluidigm	**201146A**		
Maxpar X8 antibody labelling kit, 148Nd			Fluidigm	**201148A**		
Maxpar X8 antibody labelling kit, 152Sm			Fluidigm	**201152A**		
Maxpar X8 antibody labelling kit, 155Gd			Fluidigm	**201155A**		
Maxpar X8 antibody labelling kit, 159Tb			Fluidigm	**201159A**		
Maxpar X8 antibody labelling kit, 161Dy			Fluidigm	**201161A**		
Maxpar X8 antibody labelling kit, 162Dy			Fluidigm	**201162A**		
Maxpar X8 antibody labelling kit, 167Er			Fluidigm	**201167A**		
Maxpar X8 antibody labelling kit, 170Er			Fluidigm	**201170A**		
Maxpar X8 antibody labelling kit, 174Yb			Fluidigm	**201174A**		
Maxpar X8 antibody labelling kit, 176Yb			Fluidigm	**201176A**		
*** Antibody is custom ordered without BSA or Glycerol.**						
