# Supplementary material for: MATISSE: a method for improved single cell segmentation in imaging mass cytometry
Source: BMC Biol. 2021 May 11;19:99. doi: 10.1186/s12915-021-01043-y (PMC8114487; doi:10.1186/s12915-021-01043-y)
Supplement: Supplementary file 3 — Additional file 3: Figure S1. a) Representative examples of IMC images, b) nuclear staining profiles DAPI versus Ir193, and c) predicted cell outlines of different tissues. Figure S2. a) Representative example of overlap between manual annotations and predictions, b) Recall scores calculated for different tissues, and c) Ir193 signal intensity across all analyzed images. Figure S3. Comparison of IMC and MATISSE performance per phenocluster. a) Cell numbers identified per phenocluster across all analyzed images. b) representative examples of cell outlines, density and phenoclusters, c) representative examples of cells colored by specific phenoclusters, d) fragmentation events per phenocluster. [file 12915_2021_1043_MOESM3_ESM.pdf]

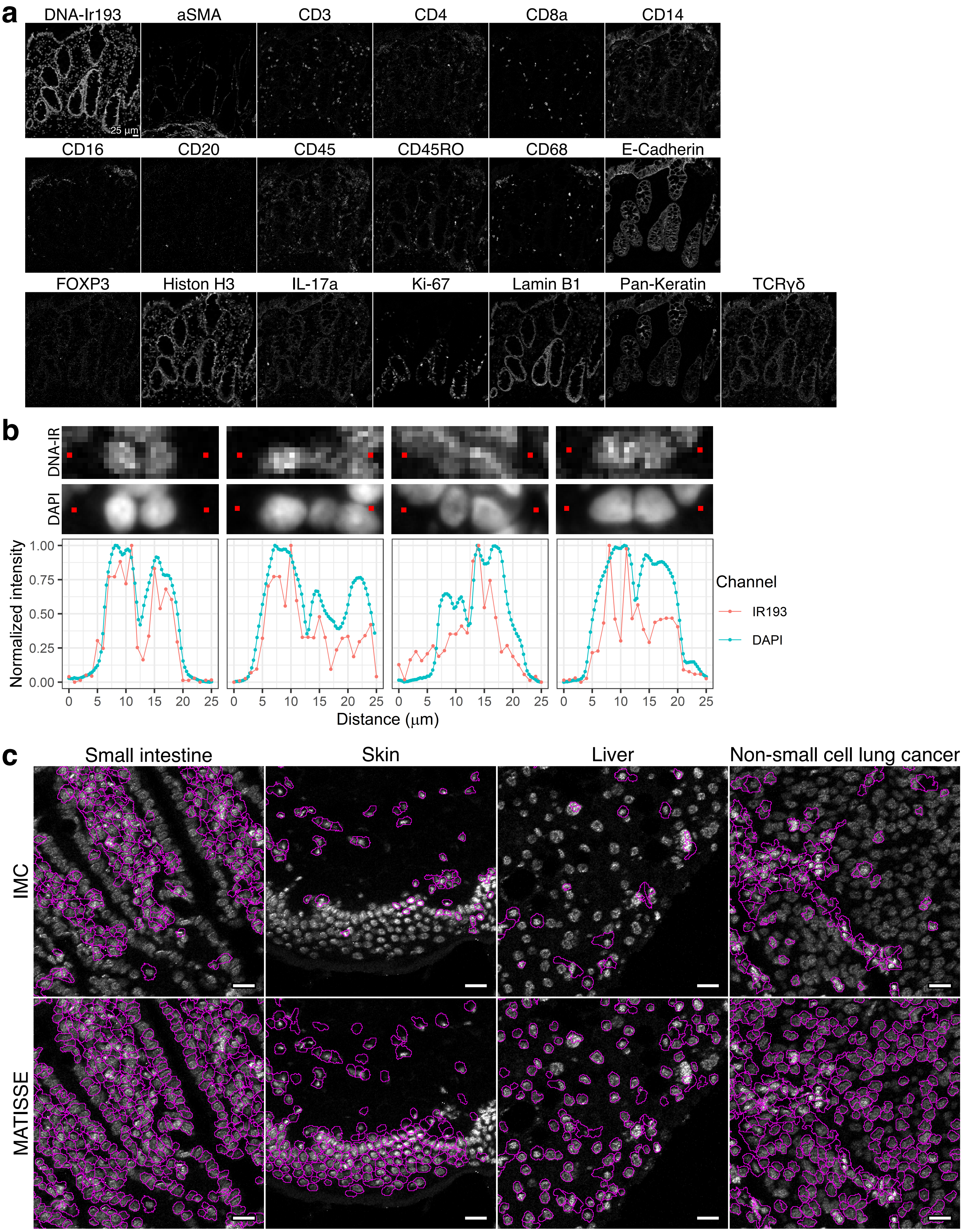

Supplementary Figure 1

a, Shown are representative images of imaging mass cytometry data from the same region.

b, Signal profile of DNA labeling with Ir193 and DAPI was analyzed in Fiji on selected neighbouring nuclei (top panel, line trace between red dots), and displayed in plots (bottom panel). Signal intensity in plots was rescaled for minimum and maximum intensity for each trace.

c, Display of regions of interest (ROI) showing an overlay of the predicted cell outlines (pink) upon IMC or MATISSE segmentation on a representative IMC image of DNA-Ir193 labeling of small intestine, skin, liver, or non-small lung cancer tissue, processed and analyzed using the same segmentation pipeline and settings as for colorectal tissue. Scale bar 25 µm.

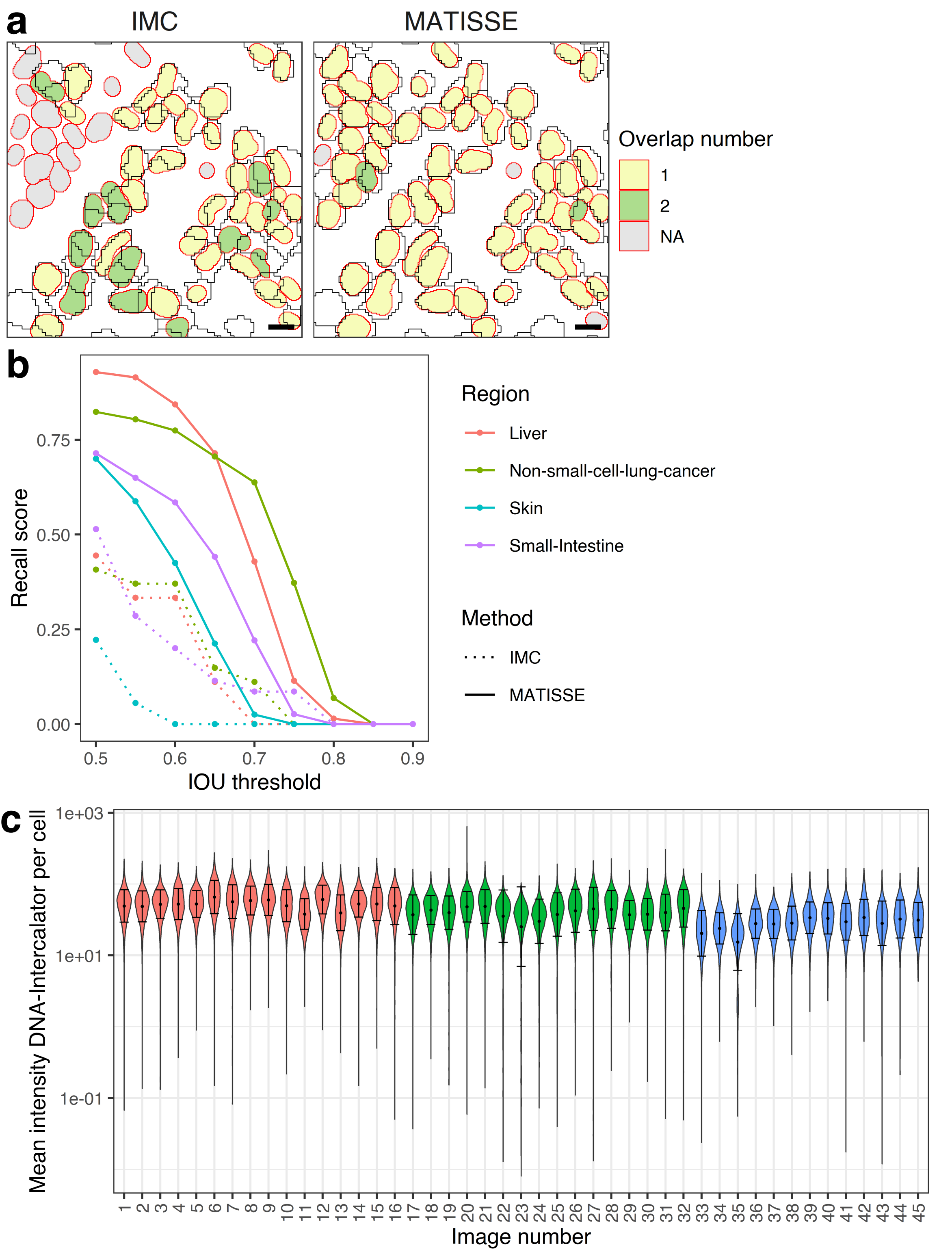

Supplementary Figure 2

a, Representative display of calculation of overlap between manually annotated events, and predicted events upon IMC or MATISSE segmentation. The number of overlap per annotated event is color coded, with no overlap (grey, NA), single overlap (yellow, 1), or 2 overlaps (green, 2), indicating split events. Scale bar 25 µm.

b, Overlap between manual annotations and predictions was quantified by recall score and compared for MATISSE and IMC at varying intersection-over-union (IOU) thresholds. n = 1 image per tissue.

c, Displayed is the mean +/- S.D. of Ir193 (DNA intercalator) signal (y-axis) per cell per ROI (x-axis), after MATISSE segmentation.

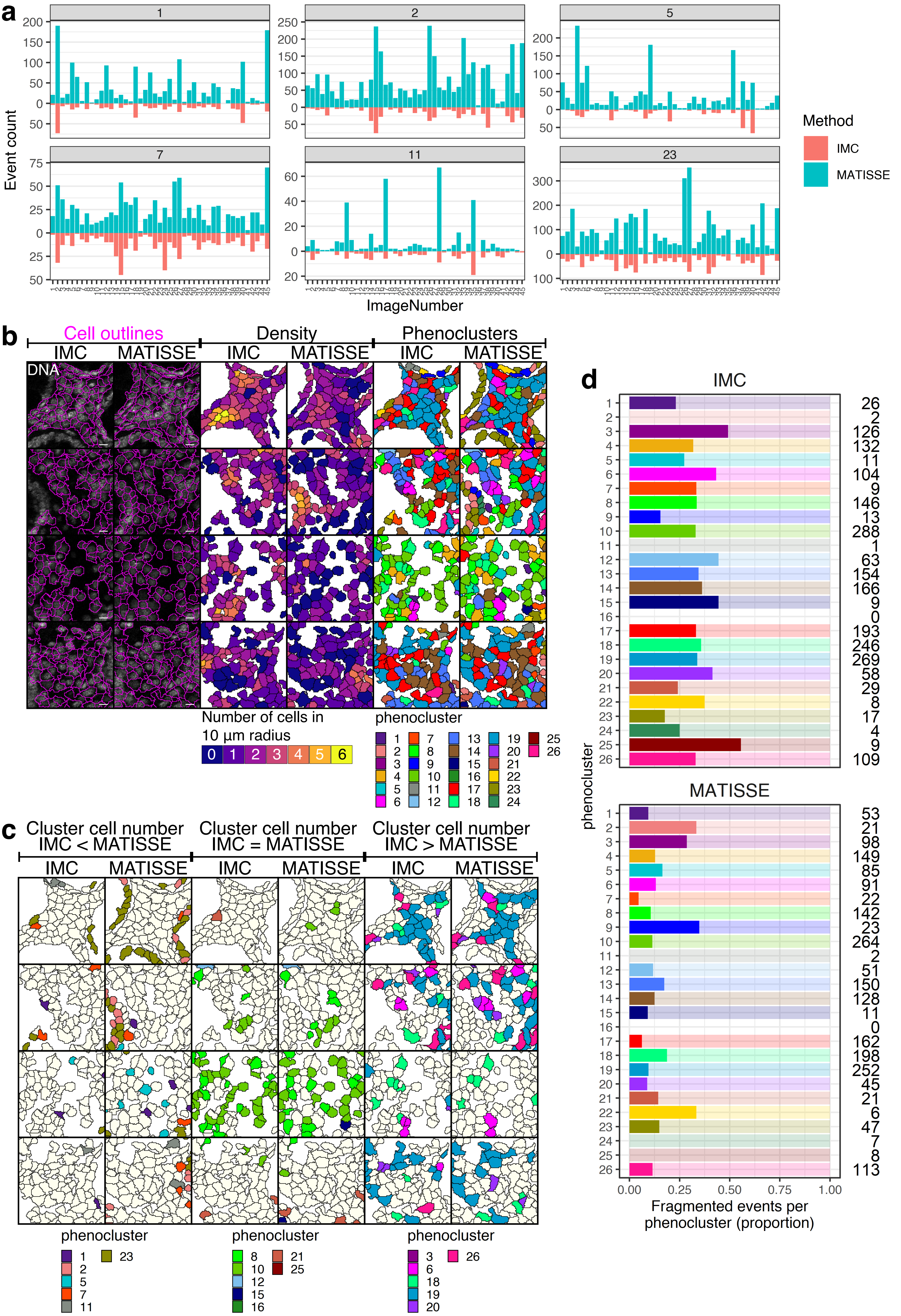

Supplementary Figure 3

a, Cell count per image (x-axis) comparing segmentation methods (MATISSE up, IMC down) for 6 phenoclusters where cell count of MATISSE larger than IMC.

b, Comparison of IMC or MATISSE segmentation of 4 randomly selected ROI's showing an overlay of the predicted cell outlines (pink) with DNA-Ir193 (white) (left columns), the color-coded display of cell density (middle), and color-coded phenoclusters (right). Cell density indicates the number of cells within a radius of 10 µm from the center of each single cell. Scale bar 10 µm.

c, Displayed highlighted phenoclusters with top 6 differential (higher numbers in MATISSE (IMC < MATISSE), lower numbers in MATISSE (IMC > MATISSE) or equal cell counts (IMC = MATISSE) comparing IMC and MATISSE.

d, Proportion of fragmented events were calculated per phenocluster, and compared between IMC (top), and MATISSE (bottom). Total number of events analyzed per cluster is indicated on the right side of the figure. N = 30 images.
