# Supplementary material for: MATISSE: a method for improved single cell segmentation in imaging mass cytometry
Source: BMC Biol. 2021 May 11;19:99. doi: 10.1186/s12915-021-01043-y (PMC8114487; doi:10.1186/s12915-021-01043-y)
Supplement: Supplementary file 4 — Additional file 4: Supplementary Table 2. Phenocluster names. [file 12915_2021_1043_MOESM4_ESM.pdf]

phenocluster	Name
1	Fibroblast
2	Epithelial
3	B-Cell
4	Fibroblast
5	-
6	T-Cell
7	Fibroblast
8	-
9	nonT, Treg, IEL
10	-
11	Epithelial, macrophage
12	Macrophage IL17a+
13	CD8+ memory T-Cell, LPL
14	Macrophage/Fibroblast/Monocyte LPL
15	CD4+, CD8+ memory T-Cell, IEL
16	γδ T-Cell/B-Cell/Monocyte
17	Macrophage, LPL
18	CD4+ memory T-Cell, LPL
19	Macrophage/T-Cell, LPL
20	Fibroblast/Macropghage LPL
21	CD8+ T-Cell, IEL
22	Regulatory T-Cell/IL-17/Monocyte
23	IEL, epithelial
24	Macrophage/Fibroblast, IEL
25	CD4+ T-Cell/Macrophage, LPL
26	Monocyte/Macrophage, LPL

Supplementary Table 2: Cluster names
